# Estimation of the Distribution of *Tabebuia guayacan* (Bignoniaceae) Using High-Resolution Remote Sensing Imagery

**DOI:** 10.3390/s110403831

**Published:** 2011-03-30

**Authors:** Arturo Sánchez-Azofeifa, Benoit Rivard, Joseph Wright, Ji-Lu Feng, Peijun Li, Mei Mei Chong, Stephanie A. Bohlman

**Affiliations:** 1 Centre for Earth Observation Sciences (CEOS) and Department of Earth and Atmospheric Sciences, University of Alberta, Edmonton, Alberta T6G 2R3, Canada; E-Mails: benoit.rivard@ualberta.ca (B.R.); jfeng@ualberta.ca (J.F.); meimei@ualberta.ca (M.M.C.); 2 Smithsonian Tropical Research Institute, P.O. Box 0843-03092, Panama City, Panama; E-Mail: wrightj@si.edu (S.J.W.); 3 Institute of Remote Sensing and GIS, Peking University, Beijing 100871, China; E-Mail: pjli@pku.edu.cn; 4 School of Forest Resources and Conservation, University of Florida, Gainesville, FL 32611, USA; E-Mail: sbohlman@ufl.edu

**Keywords:** high-resolution remote sensing, *T. guayacan*, Spectral Angle Mapping, machine learning

## Abstract

Species identification and characterization in tropical environments is an emerging field in tropical remote sensing. Significant efforts are currently aimed at the detection of tree species, of levels of forest successional stages, and the extent of liana occurrence at the top of canopies. In this paper we describe our use of high resolution imagery from the Quickbird Satellite to estimate the flowering population of *Tabebuia guayacan* trees at Barro Colorado Island (BCI), in Panama. The imagery was acquired on 29 April 2002 and 21 March 2004. Spectral Angle Mapping via a One-Class Support Vector machine was used to detect the presence of 422 and 557 flowering tress in the April 2002 and March 2004 imagery. Of these, 273 flowering trees are common to both dates. This study presents a new perspective on the effectiveness of high resolution remote sensing for monitoring a phenological response and its use as a tool for potential conservation and management of natural resources in tropical environments.

## 1. Introduction

Optical remote sensing investigations in tropical regions generally focus on the generation of land cover maps used to estimate tropical deforestation trends and habitat fragmentation at a regional and local scale [1], which in turn are used to estimate impacts on biological diversity in protected areas [2–4]. Other remote sensing monitoring efforts in tropical regions have focused on modeling ecosystem structure and composition [5,6], forest stand age [7–9], estimation of Leaf Area Index [5], Fraction of the Photochemical Active Radiation (FPAR) [10], and the identification and separability of tree species using hyperspectral imagery [11–13].

In the context of species separability using leaf and airborne spectral datasets for tropical regions, significant advances have been achieved [11–15]. Cochrane [14] using an approach designed by Price [16], illustrated the possibility of remotely identifying species using mahogany (as a reference species) and several other hardwood species from the Brazilian Amazon, but the approach was limited to an evaluation of the spectra’s amplitude and shape. Later, Clark *et al*. [11] demonstrated, using leaf spectra, that significant differences can be observed in several spectral bands across the visible and short wave infrared range for species on a tropical dry forest of Costa Rica. Castro-Esau *et al.* [12] further explored the problem of intra- and inter-species variability using a comprehensive leaf data set of tropical dry and rainforest trees from Mesoamerica. The main contribution of Castro-Esau *et al.* [12] was a novel implementation of machine learning algorithms, to better understand the potential separability among tropical tree species. Castro-Esau *et al.* [12] concluded that some level of separability exists among different species at the leaf level, and that the level of intra-species variability is sometimes as wide as the differences among distinct species. Zhang *et al.* [13] explored the same problem at the canopy level using imaging spectral data rather than single leaf measurements for a site at La Selva, Costa Rica. Zhang *et al.* [13] concluded, using the energy levels derived from wavelet coefficients, that wavelet transforms presented a robust tool for the identification of tree species using hyperspectral data, but warned that it may be impractical to expect the identification of species using only hyperspectral signals, given the high level of spectral similarity that exists at the intra- and inter-species level, confirming the finding by [12]. Similar studies at the leaf and plant level have been conducted by Tung *et al*. [17], Kamaruzaman and Ibrahim [18], Chaichoke *et al*. [19], Kelly and Carter [20] and Lucas and Carter [21]. Of importance to this work is Chaichoke *et al*. [19] whose methods diverge from established classification approaches [11], and move toward advanced classification techniques for plant species discrimination. Finally, Rivard *et al.* [15] expanded these findings to include the short wave infrared spectrum, and concluded, in agreement with Clark *et al.* [11], that significant inter-species differences exist in the shortwave infrared region of the light spectrum, and that further work is necessary to explore those linkages toward species identification in tropical regions. Furthermore, the success of single-species identification in tropical environments is also affected by the presence of liana loads and other parasitic elements living in tree crowns because of their tendency to mask the true tree spectral reflectance [22–25].

In addition to the studies mentioned above, other studies have shown the validity of using hyperspectral approaches to characterize patterns of regional ecosystem structure and function [26,27], as well as biomass estimates and biological diversity [28–30]. Although many of these studies have shown some degree of success, many of these approaches tend to be site specific, presenting problems when applied to varying ecosystems [31].

Crown-level analyses in tropical environments have benefited from the use of high resolution remote sensing (pixel <5 m), but few high-resolution studies in the tropics have been conducted, in part due to data costs. Pioneer studies have demonstrated the value of high-resolution satellite imagery for monitoring crown diameter and tree mortality in tropical environments [32–34]. The work of Asner *et al.* [32] and Palace *et al.* [34] has been especially promising regarding crown delineation/identification in tropical regions, which, in turn, can be linked to tree architecture studies. Tree crown separation is also of value in guiding the analyses of hyperspectral datasets.

One aspect that has not been explored in tropical environments is the linkage between high-resolution remote sensing, tree phenology (defined here as tree flowering events) towards species identification and mapping. Such studies have the potential of providing important information on the extent of populations of threatened or endangered tree species. In addition, in the context of long-term ecological monitoring programs, these studies may provide important insights into the response of tropical ecosystems to climate change and habitat fragmentation via the quantification of the extent of specific tree populations using reproduction signals as a proxy.

In this context, this paper explores how a combination of spectral analysis techniques applied to two Quickbird satellite images can be used to map the spatial distribution of reproductively mature *Tabebuia guayacan* trees at Barro Colorado Island (BCI) in Panama based on flowering events. The *T. guayacan* tree is a hardwood tree, used extensively since colonial times in Central America for construction projects. *T. guayacan* has one of the most extensive flowering responses to precipitation after the dry season in the tropics [35], making it an excellent candidate to evaluate the effectiveness of high resolution remote sensing techniques for mapping an explicit phenological expression (e.g., flowering episode). Such information can be used in developing conservation and sustainable management plans for this and related species.

## 2. Methods

### 2.1. Description of the Image Data and the Study Site

**Figure 1.**
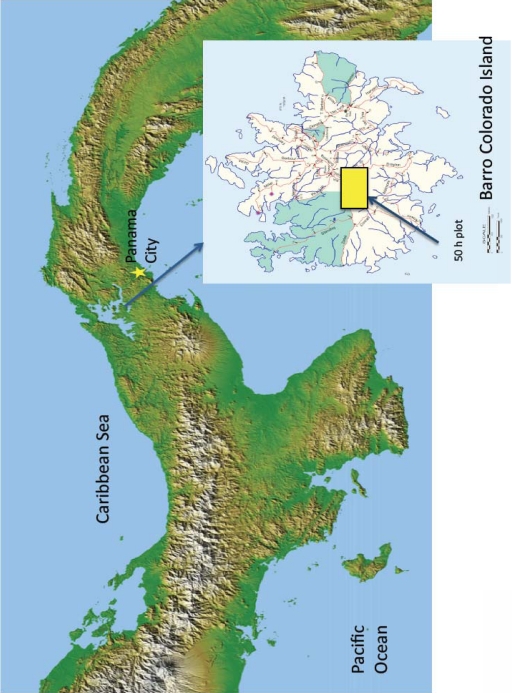
Location of Barro Colorado Island, Republic of Panama. Inset shows the location of the 50 ha plot used to validate the results of this study.

Two high-resolution Quickbird satellite images (2.4 m pixels) acquired in 29April 2002 (5% cloud cover) and 21 March 2004 (0% cloud cover) over Barro Colorado Island (BCI) in Panama (Figure 1) were used for this study. Acquisition dates were selected to capture extensive flowering events of *T. guayacan* (Bignoniaceae).

**Figure 2.**
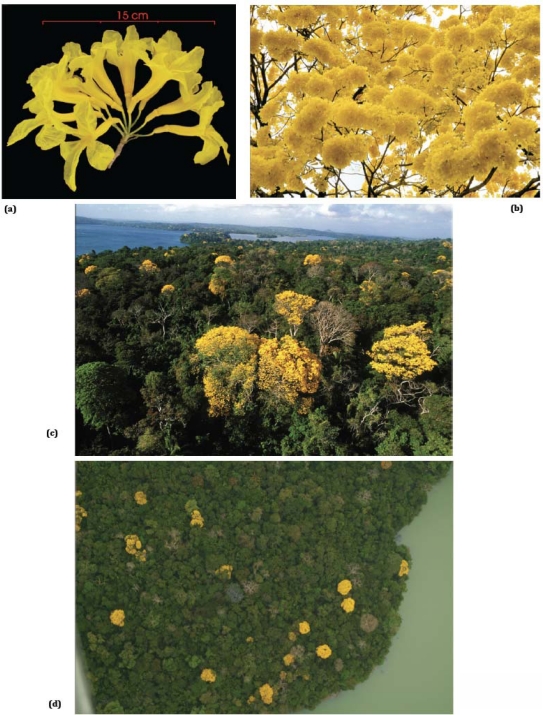
Observations from *T. guayacan* flowers **(a)** Single flower, **(b)** branch, **(c)** canopy, and **(d)** landscape level from high-resolution aerial photography.

*T. guayacan* flowering events are triggered at the end of the dry season (February–April), following short, intense precipitation episodes. *T. guayacan* flowering phases are “big bang” events characterized mostly by a *“single brief highly conspicuous burst of mass flowering.*” A *T. guayacan* canopy grouping typically presents up to 10,000 flowers in one single flowering event. Flowers can range from one to four inches in diameter, and grow in dense clusters. These flowers have a life expectancy of just two days [35]. Further studies [35] indicate that adjacent *T. guayacan* trees display near-perfect inflorescence synchronization. Flowering events may occur one or two times in a year (Figure 2).

The aforementioned phenological traits of this species make it remarkably well-suited to Quickbird image evaluation for population estimates. Two *T. guayacan* traits stand out: (1) brief inflorescence periods due to short-lived flowering bodies, and (2) synchronization of inflorescence due to precipitation triggering. The first trait centers on the short lifespan of an individual *T. guayacan* flowers, which can survive for no longer than two days, supporting the assertion that the two selected images in this study captured the entire regional phenological expression. In this context, for example, the 29 April 2002 Quickbird image captured all trees that flowered either April 28th or the 29th In other words, if all flowers were present on April 28th, all flowers will be dead and undetectable by April 30th.

The second trait that makes *T. guayacan* suitable for populations estimate by Quickbird image evaluation is synchronization. Inflorescence occurs in response to a large precipitation event, which synchronizes flowering for all adjacent individuals. Gentry [35], in documenting phenological observations of *T. guayacan* in Panama, indicated that this specific species presents “*an amazing coordination of flowering between all the individuals of a population.*” In other words, all trees observed on the Quickbird images are flowering at the same time, and they flower for two days only.

Both Quickbird images were georectified to UTM Zone 17 North. The April 2002 image was georeferenced using the Barro Colorado Island geo-spatial database and used as the master imagery for an image-to-image rectification of the March 2004 image. In both cases, a second-degree polynomial-resampling algorithm was used, given the relatively flat relief of the region. Root mean-square errors associated with the geo-rectification were estimated to be on the order of 1.5 m.

The imagery was atmospherically corrected by first converting the Quickbird data into radiance values using two different sets of absolute radiometric calibration factors (K values) provided by Digital Globe®. K values for 2002 and 2004 were different since they changed on 6 June 2003. Once that the images were converted into radiance values, an atmospheric correction using FLAASH with a tropical atmospheric model and sensor filter model was implemented using ENVI®.

To validate predictions from imagery, we used field data collected from a 50-ha long-term monitoring plot managed by the Smithsonian Tropical Research Institute (STRI, see insert in Figure 1). In this plot, STRI has identified all individuals of tree species. These data over the 50-ha plot reveal a population of 22 *T. guayacan* trees with a Diameter at Breast Height (DBH) equal to or higher than 0.20 m. Pursuing such an inventory for the entire island was not economically feasible.

### 2.2. Processing Algorithms

Image processing aimed at demarcating the centroid of flowering *T. guayacan* trees was conducted using two techniques: spectral angle mapping or SAM [39], and linear spectral unmixing, or LSU [40,41]. SAM computes the similarity of an unknown spectrum (of a pixel) in comparison to a reference spectrum, and has the advantage of allowing targeting of specific objects under variable illumination in the image [39]. LSU determines the abundance of pure endmember (class) materials within a pixel, based on the assumptions the pixels are pure and that the reflectance of each pixel is a linear combination of the reflectance components of each endmember material within the pixel. The performance of these algorithms is dependent on several factors, primarily including the data used and the spectral endmember selected [42]. Both algorithms (SAM and LSU) can be used to convert hyperspectral/multispectral imagery into biophysical information or thematic maps. Both SAM and LSU were used in this study to detect flowering *T. guayacan* trees, and the results were jointly analyzed to improve the mapping accuracy for mixed pixels. The two algorithms are similar in that both techniques require input of the target spectral signature (referred to herein as endmember), which in this case was obtained by averaging the brightest pixels of flowering *T. guayacan* trees as defined by One-Class Support Vector Machines (OCSVM).

#### 2.2.1. One-Class Support Vector Machines (OCSVM)

The detection of the yellow flowering *T. guayacan* represents a one-class classification problem, a special case of the binary (two class) classification problem where data from only one class, the *target class*, are available and sampled well. The other class, the *outlier class*, is sampled sparsely or not at all, and the boundary between the two classes must be estimated from data of the available objects. Thus, the task is to define a boundary around the target class, such that it encircles as many target examples as possible and minimizes the chance of accepting outliers.

Thus, OCSVM, a recently developed one-class classifier and also a special type of Support Vector Machine (SVM), was adopted in the present study for the specific purpose of detecting pure pixels of flowering *T. guayacan* and excluding mixed pixels and pixels of background targets. The SVM is a statistical learning method [43], which can effectively handle high-dimensional data with a limited number of training samples. The SVM has shown considerable potential for the classification of remotely sensed data [44,45]. In the two-class formulation, the basic idea is to map feature vectors to a high dimensional space and compute a hyper-plane that not only separates the training vectors from different classes, but also maximizes this separation by making the margin as large as possible.

Scholkopf *et al.* [46] developed OCSVM to deal with the one-class classification problem. The OCSVM algorithm first maps input data into a high dimensional feature space via a kernel function, and then iteratively finds the maximal margin hyper-plane that best separates the training data from the origin. The OCSVM may be viewed as a regular two-class SVM where all the training data reside in the first class, and the origin is taken as the only member of the second class. Thus, the hyper-plane (or linear decision boundary) corresponds to the classification function:

(1) f(x)=〈x,w〉+b

where *w* is the normal vector and *b* is a bias term. The OCSVM solves an optimization problem to find the function *f* with maximal geometric margin. This classification function can be used to assign a label to a test example *x*. If *f* (*x*) < 0, *x* is labeled as an anomaly, otherwise it is labeled normal.

Using kernel functions, solving the OCSVM optimization problem is equivalent to solving the following dual quadratic programming problem:

(2) $$\underset{\alpha }{\text{min}}\frac{1}{2}\sum_{i,j}\alpha_{i}\alpha_{j}K(x_{i},x_{j})$$

subject to the conditions $0\leq \alpha_{i}\leq \frac{1}{\mathrm{vl}}$ and $\sum_{i}\alpha_{i}=1$where *α_i_* is a Lagrange multiplier, which can be thought of as a weight for example *x*, and *ν* is a parameter that controls the trade-off to maximize the distance of the hyper-plane (or hyper-sphere) from the origin (background data) and also maximize the number of data points (target data) contained by the hyper-plane; *l* is the number of points in the training dataset, and *K*(*x_i_,x_j_*) is the kernel function. By using the kernel function to project input vectors into a feature space, nonlinear decision boundaries are allowed. Some commonly used kernels are linear, polynomial, and Gaussian radial basis function (RBF) kernels.

In the present study, we used the LIBSVM 2.85 [47] algorithm available at *http://www.csie.ntu.tw/~cjlin/libsvm* for the extraction of the flowering *T. guayacan* trees for each year. LIBSVM is an integrated tool for support vector classification and regression that implemented Sholkopf’s algorithm for a one-class SVM. We used the default RBF kernel for a one-class SVM.

Two parameters were selected in the classification: *ν* (*Nu*) and *γ*. The parameter *ν* (*Nu*), as described above, they perform a trade-off for allowing more target pixels inside the description and making the description more general for separating targets from the background. This is an important setting that also makes the algorithm tolerant to noise that might be present in the training set, which can be determined from ground truth data, or visually identified from image data (in this study). The parameter *γ* is the parameter from the RBF kernel, controlling the width of the Gaussian kernel [48].

For each of the 2002 and 2004 images, the output is a binary map locating pure pixels in the scene. In each case, the spectra of the pixels identified within this map were averaged in order to obtain a representative *T. guayacan* endmember spectrum. The latter was then used as an input for the SAM and LSU analysis described below.

#### 2.2.2. SAM and LSU

The Spectral Angle Mapper (SAM) calculates the spectral angle (*θ*) between a target spectrum (*t⃗*) and the spectrum of each pixel (*r⃗*) of the image by:

(3) $$\theta =\;{\text{cos}}^{-1}((\vec{t}•\vec{r})/(\left|\vec{t}\right|•\left|\vec{r}\right|))$$

This method treats both spectra as vectors. The value of the spectral angle is insensitive to illumination, since the SAM algorithm uses only the vector direction and not the vector length.

Linear spectral unmixing (LSU) analysis is based on the premise that most pixels in an image are a mixed response of a set of linearly independent endmember spectra. To deconvolve a spectrum into fractional abundances of its constituent endmembers, the following equation can be solved using a least squares approach:

(4) $$r_{b}=\sum_{i=1}^{n}F_{i}R_{\mathrm{ib}}+E_{b}$$

where ***r_b_*** is the reflectance of the pixel at band *b*, ***F_i_*** is the fractional abundance of the endmember *i*, ***R***_***i***b_ describes the reflectance of endmember *i* at band *b, n* equals the number of endmembers, and ***E_b_*** is the error of the least square fit at band *b*. The results of LSU are fractional abundances of the endmembers in each pixel.

**Figure 3.**
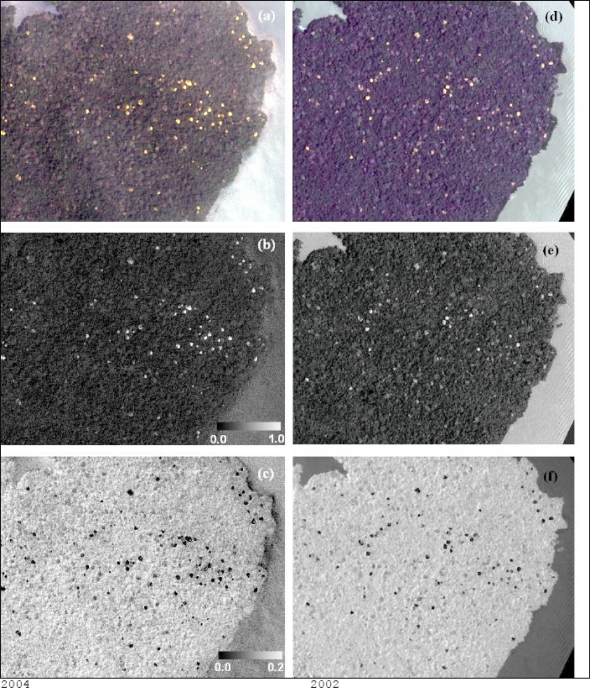
Selected area of Barro Colorado Island Quickbird satellite imagery (northwest region) of flowering *T. guayacan* acquired in **(a)** March 21st, 2004 and **(d)** April 29th, 2002. **(b–e)** Abundance map of *T. guayacan* estimated from the LSU algorithm. Scale bar represents the range of per-pixel fractional abundance. **(c–f)** Result from SAM algorithm for the same region. Scale bar represents the range of spectral angle (in radian) between the spectrum of each pixel and that of *T. guayacan.*

**Figure 4.**
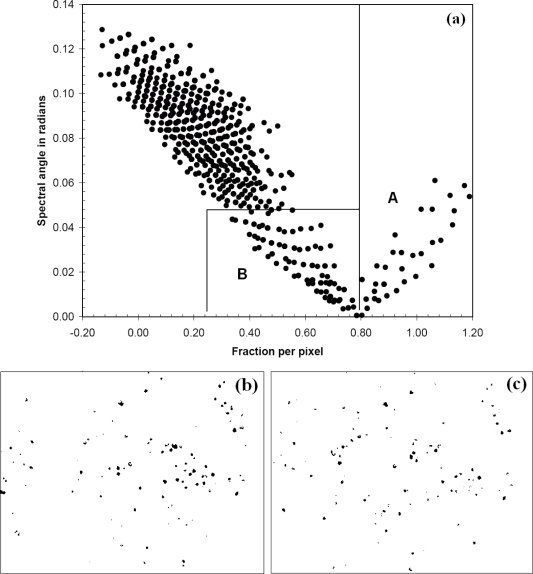
**(a)** Scatter plot of *T. guayacan* fractional abundance and spectral angle for each pixel of the 2004 data for the area shown in Figure 3. Region A (fraction > 0.80) on the plot represents the purest pixels of flowering *T. guayacan* tree. Region B (0.25<fraction<0.80 and spectral angle <0.05) represents partially mixed pixels with flowering *T. guayacan* tree. **(b)** and **(c)** display all pixels encompassed by scatterplot Region A and B for 2004 and 2002 respectively.

For both SAM and LSU, flowering portions of *T. guayacan* trees were considered the target endmember and other land covers (including generic vegetation, water, soil and man-made objects) were included in the spectral analysis as background information. Pixel spectra displaying a small spectral angle value (SAM results) represent pixels with spectra similar in shape to that of the endmember target, while pixels with larger target fractional abundances (LSU results) represent pixels that contain larger target abundance. Each analysis produced a new image indicating the presence of trees with flowers (Figure 3). These two images were then analyzed jointly to retain only pixels below a threshold spectral angle and above a threshold fractional abundance (Figure 4a). The output was a single image per date retaining pixels of flowering *T. guayacan* trees. Selection of the threshold values is discussed in the results section.

Using ERDAS Imagine 9.2 ©, both images were classified using the unsupervised classification method to identify the flowering individuals given the strong contrast observed on Figures 3c,f. Here the ISODATA clustering algorithm was used to cluster the pixels into 25 classes. Given the resolution of the imagery and the distinctiveness of the flowering individuals (Figures 3c,f), it was determined that 25 classes were more than sufficient to isolate the pixels that represented the flowering individuals. Once identified, the data were exported and added to a GIS where the XTools Pro extension in the ArcMap module of the ESRI ArcGIS 9.2 software suite was used to convert the objects representing the flowering individuals into centroid points. The Feature Conversion used was the Shapes to Centroids operation within the XTools Pro extension. Once centroids were determined, it became apparent that some tree crowns adjacent to one another were appearing as a singletree crown, and were each represented by a single, communal centroid. In order to check for this problem, we estimated—upon of measuring several crowns diameters on the 2000 and 2004 QuickBird images—that a buffer zone of 5 m could be safely used to check against the possibility the error mentioned above. As such a two-step quality control process was used. First, every centroid was automatically checked using a buffer analysis (buffer = 5.0 m ArcGIS 9.2). If two or more centroids fell within the selected range, they were considered one single crown. Second, given the high resolution of the data, manual visual inspection of all centroids was conducted by overlapping the result of the buffer analysis on top of a false color composite (RBG) of each QuickBird image. Though a subjective process, this task was easily performed, as multiple tree crowns appeared as notably irregular circles easily distinguishable from the circular shape of single, stand-alone tree crowns. Furthermore, given the relatively small size of the study area, this was a surmountable task done by visual inspection by an experienced analyst. The resultant centroid map product allowed for a count of flowering trees.

#### 2.2.3. Accuracy Assessment and Cartographic Analysis

Since neither field nor aerial photography information is currently available for the years on which the QuickBird datasets were acquired, and therefore preventing the implementation of a conventional error analysis using a contingency matrix; we used two approaches to evaluate how well our algorithm detected the flowering crowns.

The first approach was ecological in nature. This approach was aimed to compare the number of reproductive trees, detected from the satellite image analysis, against those trees above a given reproductive threshold. The former information was obtained from the 50 ha Smithsonian Tropical Research Institute permanent monitoring plot [35]. This plot is currently containing a population of 22 *T. guayacan* trees with a Diameter at Breast Height larger than 0.20 m.

**Figure 5.**
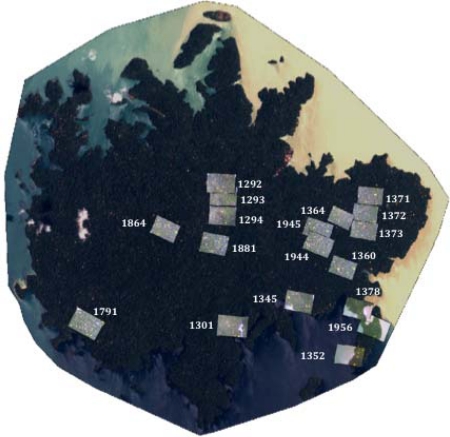
Location of 18 aerial photographs used to evaluate the accuracy of the technique. Numbers next to the aerial photography represents a unique ID assigned by the Smithsonian Tropical Research Institute (STRI). Images were acquired April 5–6, 2005. Nominal resolution is 0.20 m.

The second approach used aerial photographs acquired in 5–6 April 2005 as a reference to evaluate the success of our algorithm to detect individual flowering trees of *T. guayacan* for eighteen field sites. It is important to address here that this approach was also used to investigate the strong dynamic nature of this ecosystem where trees are growing and dying. The nominal resolution of the aerial photographs is 0.10 m. Figure 5 shows the locations and coverage of the photos used in this study. Centroids from flowering trees were visually identified for each photo and compared to the number found for the same area in both the 2002 and 2004 Quickbird images (Table 1). In addition to recording the number of flowering trees present, the number of matching individuals between both Quickbird images to the aerial photos was also recorded. This information can be used to partially illustrate the accuracy of the technique applied in this study to identify flowering trees, given the impossibility of conducting a traditional contingency table approach because of the lack of concurrent field information.

By means of change detection analysis in ArcGIS® we also determined the number of flowering crown centroids common to the 2002 and 2004 images. This process assumes that centroids common to the two years represent the same trees, while other non-overlapping centroids between the two years represent tree crowns that flowered in 2002, but not in 2004. In the latter assumption, two scenarios are possible: (1) If the centroid is present in 2002, but not in 2004, the tree represented by that centroid has likely fallen, died or simply did not reproduce during the 2004 flowering event; (2) If the centroid is present in 2004 but not in 2002, the tree represented by that centroid is likely a new growth, meaning that it reached a DBH that allowed it to reproduce. Once anomalous fallen and new growth trees had been identified, the number of individuals overlapping both years was tallied up to yield the total number of common trees between the two years.

## 3. Results

Results from the application of the OSCVM technique indicate two sets of parameters for the accurate extraction of pure *T. guayacan* pixels. For the 2002 dataset *ν* (*Nu*) and *γ* were estimated to be 0.1 in both cases. For the 2004 dataset *ν* (*Nu*) and *γ* were estimated to be 0.1 and 7.5 respectively.

For each year, the output from OSCVM was treated as a pure *T. guayacan* endmember input for the LSU and SAM analysis. Then, a plot of flowering *T. guayacan* fractional abundance *vs.* spectral angle was generated for the 2002 and 2004 image (Figure 4a). For both datasets, the purest pixels dominated by flowering *T. guayacan* tree (more than 80% in fractional abundance per pixel) were identified using a threshold of *F* > 0.80 (Region A, Figure 4a). Mixed pixels partially occupied by flowering *T. guayacan* tree (25%∼80% in fractional abundance per pixel) were mapped by combining fractional abundance (0.25 < *F* < 0.80) and spectral angle values (<0.05 in radian) (Region B, Figure 4b,c), and, in general, they represent the edges of crowns.

**Figure 6.**
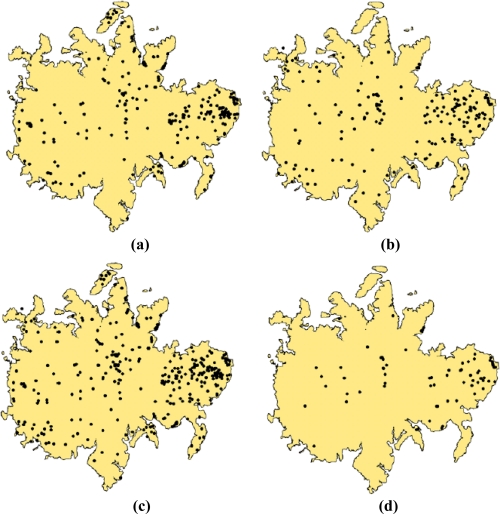
Distribution of *T. guayacan* extracted using a target abundance threshold >80% for **(a)** April 29, 2002; **(b)** March 21st, **(c)** Combined populations excluding overlapping trees, and **(d)** trees common to the two dates.

The information generated from these two approaches is presented in Figures 3c,f and *T. guayacan* trees in flower during the two events are easily identifiable in comparison to their background. As such, our post-classification analysis using a centroid-based GIS approach clarified by a minimum clearing of overlapping crowns was able to detect 422 trees [Figure 6(a)] in the 2002, and 557 trees in 2004 [Figure 6(b)]. Two hundred and seventy three flowering trees were identified as common to imagery from both years (Figure 6c,d). Two clusters of trees are observed in Figure 6c. A large cluster is located in the eastern portion of the island and smaller cluster is located in the central section of the island. Besides these two areas, the remainder of the trees tends to be randomly distributed across the island without a noticeable geographic pattern.

Of the 22 trees identified in the 50-ha plot estimated to have a DBH larger than 0.20 m, only eight and five were identified with flowers in the 2002 and 2004 imagery, respectively. Four of these trees were identified with flowers in both years, and 13 were not identified with flowers in either year. Visual inspection of the images showed no evidence that these trees had flowers. It is most likely that these trees failed to flower during both flowering events. All 13 have DBH larger than the reproductive size threshold of *T. guayacan* (0.3 m); however, the proportion of trees flowering increases gradually with DBH for tree species on Barro Colorado Island [50]. Therefore, we cannot determine whether the trees failed to flower in both years or flowered and failed to be detected.

Comparing the aerial photography with the outcome of our remote sensing approach, we estimated that 88% of the trees detected in the 2002 images were also detected in the 2005 aerial photographs (Table 1). Less (73%) of the crowns detected in 2004 were also detected in the 2005 aerial photographs. However, conducting a normal accuracy assessment based on a contingency table approach was not possible given the flowering dynamics of this species. This is illustrated by image 1372 in Table 1. For this image 13 trees were identified having flowers in both for 2004 and 2005. Although the number of trees is the same both years, which suggests 100% accuracy between both years, only 9 of the same trees were common in 2004 and 2005. This does not mean that we have a higher error in the classification but most likely that different trees were flowering during these years.

## 4. Discussion

High-resolution remote sensing imagery has proven extremely useful to identify tree crown boundaries in tropical environments, and to investigate the relationship between tree mortality, crown diameter, and shape [31–34]. However, the application of high-resolution multispectral imagery to identify tree populations using phenological expressions as a proxy had not been explored until now. The identification of the potential extent of tree populations using high resolution remote sensing can be facilitated if a plant’s natural history and phenological expressions are integrated into a regular remote sensing monitoring process. In this specific case, our understanding of when the trees will flower, combined with observations regarding the duration of the flowering event, the inflorescence synchronization common to this species, and the lifespan of the thousands of flowers produced by the *T. guayacan* [35], combined with ecological theory [49,50] allows us to identify the potential extent of the population of this tree if it has reached a given reproductive threshold.

The fact that only 273 *T. guayacan* trees were common to both blooming events, and that the total number of flowering trees identified in each image (422 in 2000 *vs.* 557 in 2004) differed substantially, poses important monitoring questions regarding frequency of data acquisition for studies aimed to monitor and assess tropical biodiversity using high resolution remote sensing. Specifically, our study demonstrates that the process of data acquisition may require several multi-temporal collections over several years, as well as an in-depth knowledge of the eco-physiological process driving flower production. Another important element to be considered is the presence or absence of lianas in the canopy that can affect our ability to use natural history to define and implement programs for the detection of tree populations that are reproducing. Wright *et al*. [50] have indicated that the presence of lianas is one of the key variables affecting tree reproduction even if such trees have reached the threshold reproductive DBH. In fact, [50] clearly indicated that liana loads influence the size-dependent probability of reproduction for specific tree species, and the presence of lianas may potentially factor highly in the differences observed in the number of trees detected in both years in this study.

Our study documents these issues (temporal acquisition and eco-physiological knowledge) showing that despite using selective targeting and ecological knowledge of flowering patterns for the *T. guayacan* tree, not all flowering trees in a given year were identified. The fact that only thirteen of the twenty-two *T. guayacan* trees present at the Smithsonian Tropical Research Institute’s permanent 50-ha plot were detected by spectral analysis focusing on the presence of flowers demonstrates the difficulty of using remote sensing observations to estimate the total number of individuals just based on limited observations of flowering episodes, stressing the need to have a good understanding of not only the natural history of the tree under consideration, but also those elements that affect its reproduction (e.g., liana loads).

**Figure 7.**
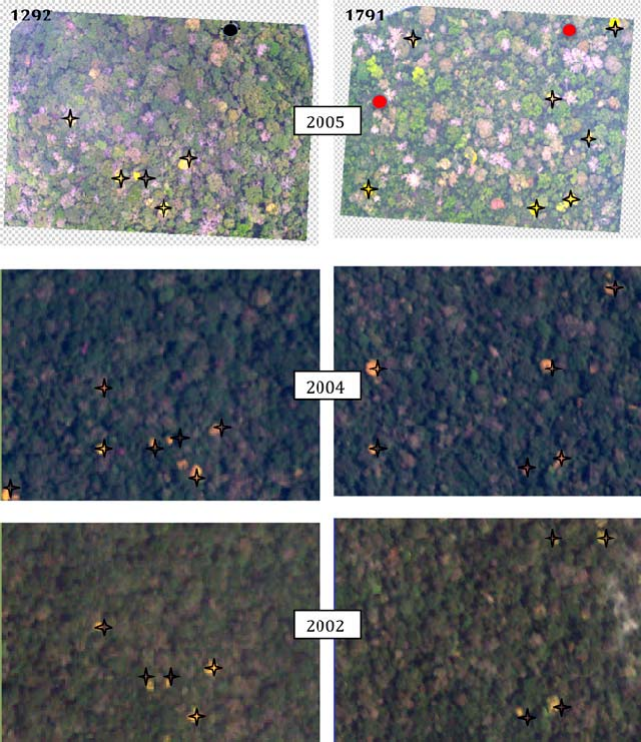
Comparison of Quickbird satellite images for 2002 and 2004 and the Smithsonian Tropical Research Institute’s aerial photography (1292 and 1791). Starts represent detected flowering trees (images and photos), black dots represent a tree with new leaves and red dots represent dead trees in 2005 that flowered in 2004 and 2002.

Given that the total population of *T. guayacan* for the whole BCI Island is unknown and logistically impossible to estimate, it is difficult to assess whether the tree counts provided in this study provide a total estimate of the overall population of this species at BCI. This was not the primary goal of our paper. Figure 7 shows the difficulty on estimating even the same trees from year to year, which prevents the possibility of conducting traditional accuracy assessments. What we can indicate for sure is that our approach has been successful in identifying the majority of those trees that are above a given reproduction threshold. Even though the census of individuals is not complete, the derived maps can provide important information on the relative densities of species [51] or provide the basis of modeling of the extent and timing of flowering [52]. Wright *et al.* [50] define reproductive size threshold as the inflection point of the relationship between DBH and the probability of an individual to be reproductive (e.g., flower production). Wright *et al.* [50] also state that in the context of tropical tree species, the relative size of onset of maturity (RSOM) is a function of a given DBH and it contributes to species coexistence at a given site. Therefore, the issue is not the fact that we can identify every single tree from this species in the landscape, but that we can identify the fraction of the population that is reproducing. This has significant implications for the ecology and conservation biology of *T. guayacan*, a key species, along with other trees such as *D. panamensis,* for the survival of the Great Green Macaw *(Ara ambiguus)*. Identifying the whole population could be relevant to tree counting and other compositional studies, but the full identification of trees that are reproducing provides significant insights on the future viability of the population of this important tropical tree, and opens new doors to the conservation biology community to consider how targeted remote sensing observations can provide important insights into their conservation efforts in tropical environments.

## 5. Conclusions

Our study suggest that studies aimed at estimating the spatial distribution and total population size of a given species based on massive flowering events or *big bang* events must be handled with great care. High-resolution remote sensing images and their processing using advanced classification approaches, such as the ones used in this paper, can provide approximations of the presence or absence of a given tree species within a specific region that (a) has a DBH above a given reproduction thereshold and (b) have massive and synchronized phenological expressions. Our work also stresses the fact that if a species is present it does not means that it can be identified based on phenological expressions (e.g., flower productions) since in many cases these species may not be old enough to allow for such expression, or may be infested by lianas which hinders it reproduction. Therefore the approach presented here should be used not from the point of view of just simply counting trees, but in the context of obtaining a potential idea of the overall number of reproductive trees present in a region. The former has more significant impact towards biodiversity conservation that just tree identification and counting their number.

Furthermore, while the use of a combination of high-resolution remote sensing and classification algorithms during flowering events may provide important insights into the spatial distribution of a species and help to define clusters for potential genetic studies, it cannot replace field campaigns aimed at mapping the large-scale distribution of species. The approach presented here can be used as a first approach toward defining sampling areas in a given region. In addition, the use of high-resolution imagery and the ecological knowledge of flowering patterns have the potential to serve as a powerful tool for the identification and cataloguing of members of this species using emerging hyperspectral airborne remote sensing platforms.

## Figures and Tables

**Table 1.** Intercomparison of results between computer-derived crowns and aerial photography interpretation of flowering trees. Number on parenthesis represents trees that did not flower in 2005 but that did flower in 2002 and 2004.

**Aerial** **Photo ID**	**2002** **Quickbird image**	**2004** **Quickbird image**	**2005** **air photo**	**Matching trees** **2002–2005**	**Matching trees** **2004–2005**
1292	5	7	6	5	5 (−2)
1293	8	8	9	7 (−1)	7 (−1)
1294	8	14	9	8	9 (−5)
1301	3	6	4	3	3 (−3)
1345	3	6	7	3	6
1352	2	6	8	2	6
1360	3	5	3	3	3 (−2)
1364	7	8	8	7	5 (−3)
1371	3	8	7	2 (−1)	6 (−2)
1372	9	13	13	8 (−1)	9 (−4)
1373	6	13	9	3 (−3)	4 (−9)
1378	3	4	7	3	3 (−1)
1791	4	6	7	3 (−1)	5 (−1)
1864	2	3	7	1 (−1)	2 (−1)
1881	3	3	9	3	3
1944	15	11	12	11 (−4)	6 (−5)
1945	10	16	15	10	9 (−7)
1956	2	6	6	2	6
